# The Potential Role of Seaweeds in the Natural Manipulation of Rumen Fermentation and Methane Production

**DOI:** 10.1038/srep32321

**Published:** 2016-08-30

**Authors:** Margarida R. G. Maia, António J. M. Fonseca, Hugo M. Oliveira, Carla Mendonça, Ana R. J. Cabrita

**Affiliations:** 1 REQUIMTE, LAQV, ICBAS, Instituto de Ciências Biomédicas de Abel Salazar, Universidade do Porto, Rua de Jorge Viterbo Ferreira n.° 228, 4050-313 Porto, Portugal; 2 REQUIMTE, LAQV, DGAOT, Faculdade de Ciências, Universidade do Porto, Rua do Campo Alegre s/n, 4169-007 Porto, Portugal; 3 ICBAS, Instituto de Ciências Biomédicas de Abel Salazar, Universidade do Porto, Rua de Jorge Viterbo Ferreira n.° 228, 4050-313 Porto, Portugal; 4 CECA, Centro de Estudos em Ciência Animal, Universidade do Porto, Campus Agrário de Vairão, Rua Padre Armando Quintas, 4485-661 Vairão, Portugal

## Abstract

This study is the first to evaluate the effects of five seaweeds (*Ulva* sp., *Laminaria ochroleuca*, *Saccharina latissima*, *Gigartina* sp., and *Gracilaria vermiculophylla*) on gas and methane production and ruminal fermentation parameters when incubated *in vitro* with two substrates (meadow hay and corn silage) for 24 h. Seaweeds led to lower gas production, with *Gigartina* sp. presenting the lowest value. When incubated with meadow hay, *Ulva* sp., *Gigartina* sp. and *G. vermiculophylla* decreased methane production, but with corn silage, methane production was only decreased by *G. vermiculophylla*. With meadow hay, *L. ochroleuca* and *S. latissima* promoted similar methane production as the control, but with corn silage, *L. ochroleuca* increased it. With the exception of *S. latissima*, all seaweeds promoted similar levels of total volatile fatty acid production. The highest proportion of acetic acid was produced with *Ulva* sp., *G. vermiculophylla*, and *S. latissima*; the highest proportion of butyric acid with the control and *L. ochroleuca*; and the highest proportion of *iso*-valeric acid with *Gigartina* sp. These results reveal the potential of seaweeds to mitigate ruminal methane production and the importance of the basal diet. To efficiently use seaweeds as feed ingredients with nutritional and environmental benefits, more research is required to determine the mechanisms underlying seaweed and substrate interactions.

Dietary nutrients are fermented in the rumen by a complex microbial population, producing volatile fatty acids (VFA), hydrogen, and carbon dioxide as the main fermentation products. Methane production results from the reduction of carbon dioxide with hydrogen by archaea, a group of methanogens frequently associated with ciliated protozoa^1^. Enteric methane production prevents increases in hydrogen pressure, which could inhibit the normal functioning of microbial enzymes and impair rumen fermentation^2^. Methane is a potent greenhouse gas and may represent a loss of 2–15% of the gross energy (GE) in the feed, depending on the diet^3^. Therefore, enteric methane mitigation may have a positive impact on feed utilization, diet digestibility, and, ultimately, livestock productivity^4^.

Seaweeds might be a natural alternative for the mitigation of greenhouse gas emissions by ruminants. Seaweeds have been used to feed livestock from time immemorial in coastal regions during periods of feed scarcity^5^. Renewed interest has emerged during recent decades in the use of seaweeds as feed ingredients due to their richness in organic minerals, complex carbohydrates, proteins and low-molecular-weight nitrogenous compounds, lipids, vitamins, volatile compounds, pigments^6^ and bioactive substances with broad biological activities^7^. Based on availability and market cost, seaweeds have been evaluated as a prebiotic promoter^8^ or a feed ingredient^9^ at low or high inclusion rates, respectively. In this context, due to the chemical diversity and complexity of polysaccharides, which may account for 25–75% of algae dry weight^10^, ruminants seem to be the most suitable animals to be fed on seaweeds. The intricate rumen ecosystem might provide the ruminant the ability to use seaweeds by breaking down the complex polysaccharides. Additionally, some seaweeds and seaweed extracts effectively reduce ruminal methane production *in vitro* (*e.g*., *Asparagopsis taxiformis*)^11^,^12^,^13^,^14^,^15^, although a broad range of results have been reported and with variable effects on rumen fermentation.

Notwithstanding the potential effect of the basal diet on the effectiveness of the compound used to mitigate methane emissions^16^, earlier research did not evaluate seaweed effects across more than one feed type. Therefore, the objective of this study was to determine the effects of seaweeds naturally occurring at the Atlantic and Mediterranean coasts (*Laminaria ochroleuca* and *Gigartina* sp.) or produced in an integrated multi-trophic aquaculture (IMTA) system (*Ulva* sp., *Saccharina latissima* and *Gracilaria vermiculophylla*) on the *in vitro* ruminal fermentation parameters, total gas production and methane production for two feed substrates (meadow hay and corn silage). As far as we know, this is the first report on the effects of these seaweeds on *in vitro* rumen fermentation across different substrates.

## Results

### Chemical composition

The chemical composition of the base substrates and the five seaweed species is presented in Table 1. The meadow hay and corn silage presented 723 and 493 g kg^−1^ dry matter (DM), and 565 and 377 g kg^−1^ neutral detergent fibre (NDF, DM basis), respectively. The chemical composition of the studied seaweeds showed a wide variation, particularly with respect to ash and NDF contents, which respectively ranged from 171 g kg^−1^ DM in *S. latissima* to 348 g kg^−1^ DM in *Gigartina* sp. and 71.1 g kg^−1^ DM in *S. latissima* to 335 g kg^−1^ DM in *Ulva* sp. *Laminaria ochroleuca* and *Ulva* sp. presented the highest acid detergent lignin (ADL) contents. Overall, seaweeds were poor sources of lipids, the highest content being found in *S. latissima* (7.87 g kg^−1^ DM), with GE ranging from 9.51 MJ kg^−1^ DM in *Gigartina* sp. to 12.8 MJ kg^−1^ DM in *S. latissima*. The three seaweeds cultivated in an IMTA system (*Ulva* sp., *S. latissima*, and *G. vermiculophylla*) presented the highest crude protein (CP) content.

### Total gas and methane production

Total gas and methane production were strongly affected by the basal substrate (meadow hay or corn silage) and inoculum (adapted to 0% or 5% sunflower oil) used in *in vitro* incubations and by seaweed inclusion (Tables 2 and 3).

While the use of corn silage as a basal substrate increased total gas production (*P* < 0.001) after 24 h incubation, gas production was decreased by oil-adapted inoculum (*P* < 0.001; Table 2). Overall, seaweeds led to 17% less gas production than the control (101 mL g^−1^ DM *vs*. 83.1 mL g^−1^ DM, respectively; Table 3). Among seaweeds, the red algae *Gigartina* sp. had the lowest gas production of all species, producing a total of 67.5 mL g^−1^ DM after a 24 h incubation, while the others promoted similar gas production (respectively, 82.5, 86.2, 89.5, and 89.9 mL g^−1^ DM for *G. vermiculophylla*, *Ulva* sp., *L. ochroleuca* and *S. latissima*) that was still lower than the control (101 mL g^−1^ DM).

Similar to total gas production, methane production was increased by corn silage (*P* < 0.001) and by oil-unadapted inoculum (*P* < 0.001; Table 2). Seaweeds strongly affected methane production (*P* < 0.001), a reduction in methanogenesis being observed with *Ulva* sp., *Gigartina* sp., and *G. vermiculophylla* in comparison with the control, *L. ochroleuca* and *S. latissima* (Table 3). No significant relationships were observed between gas or methane production and the chemical composition of the studied seaweeds, except that ash tended to be negatively correlated with gas production (r = −0.847; *P* = 0.070).

**Figure 1:**
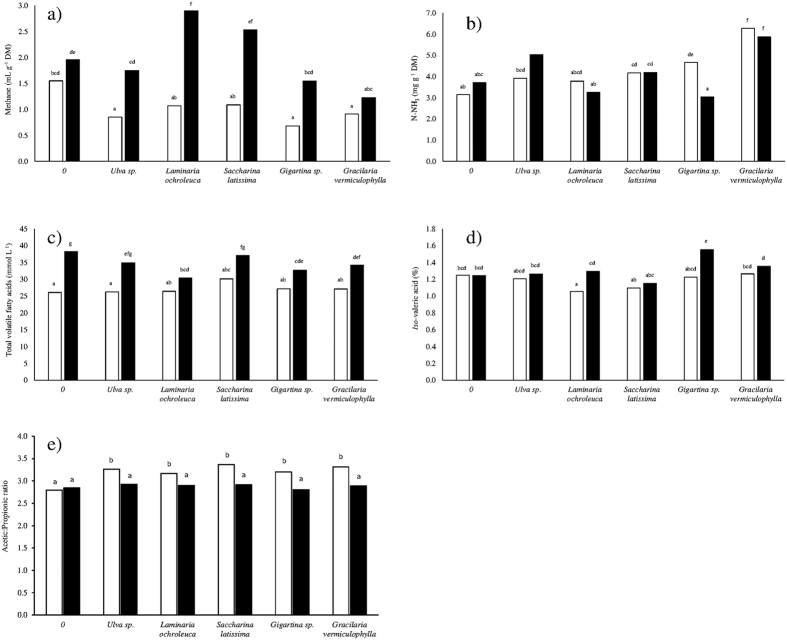
Effects of seaweed*substrate interaction on methane production [mL g^−1^ DM (**a**)], N-NH_3_ [mg g^−1^ DM (**b**)], total volatile fatty acid (VFA) production [mmol g^−1^ DM (**c**)], the *iso*-valeric proportion [% (**d**)], and the acetic:propionic acid ratio (**e**) after 24 h of *in vitro* incubation. Meadow hay (□), corn silage (■). Mean values with different superscript letters were significantly different (*P* < 0.05).

A significant interaction between substrate and seaweed was observed for methane production (Fig. 1a). When incubated with meadow hay, *Ulva* sp., *Gigartina* sp. and *G. vermiculophylla* decreased methane production to 55, 44 and 59% of the control, respectively. However, when these same seaweeds were incubated with corn silage, only *G. vermiculophylla* decreased methane production, to 63% of the control. *Laminaria ochroleuca*, and *S. latissima* promoted similar methane production as the control when incubated with meadow hay, but when incubated with corn silage, *L. ochroleuca* increased methane production to 148% of the control.

### Fermentation pH and ammonia-N production

Fermentation pH was strongly affected by basal substrate (*P* < 0.001; Table 2) and tended to be affected by inoculum (*P* = 0.056; Table 2), while seaweed had no effect (*P* = 0.306; Table 3). Incubation of meadow hay led to a higher fermentation pH compared to corn silage, and oil-adapted inoculum tended to decrease pH.

Ammonia-N production (mg g^−1^ DM) was unaffected by substrate (*P* = 0.406) but was higher (*P* < 0.001) in oil-unadapted inoculum than inoculum adapted to 5% oil supplementation (Table 2). Overall, ammonia-N (NH_3_-N) production was increased by nearly 30% with seaweed inclusion compared to the control (4.42 *vs*. 3.43 mg g^−1^ DM, respectively; *P* < 0.001; Table 3). Among seaweeds, the brown *L. ochroleuca* and *S. latissima* had similar NH_3_-N production as the control (3.52 and 3.86 mg g^−1^ DM, respectively), and the red algae *Gigartina* sp. had the highest NH_3_-N production (6.07 mg g^−1^ DM; Table 3).

A significant interaction between substrate and seaweed was found for NH_3_-N production (Fig. 1b). When incubated with meadow hay, the red seaweeds increased NH_3_-N production by 1.5- to 2-fold. When corn silage was the substrate, *Ulva* sp. and *G. vermiculophylla* decreased NH_3_-N production by 1.4- and 1.6-fold.

### Volatile fatty acid production and profile

Volatile fatty acid production and proportion were affected (*P* < 0.001) by basal substrate and inoculum, except valeric acid that was only affected by inoculum (*P* < 0.001; Table 2). Corn silage decreased the acetic acid proportion and the acetic:propionic ratio and increased all individual and total VFA concentrations (mmol g^−1^ DM; *P* < 0.001; Table 3). Oil-adapted inoculum increased the proportion of propionic, *iso*-butyric, and *iso*-valeric acids and total VFA but decreased the proportions of acetic, butyric, valeric and caproic acids and the acetic:propionic ratio (*P* < 0.05; Table 2). Total VFA production (mmol g^−1^ DM) was higher with *S. latissima* than with any other seaweed (*P* < 0.05; Table 3), although it did not differ from the control. Total VFA production of all the other seaweeds was also similar to the control. The interaction between substrate and seaweed showed that *L. ochroleuca*, *Gigartina* sp. and *G. vermiculophylla* tended (*P* = 0.092) to decrease total VFA production when incubated with corn silage (Fig. 1c).

The proportion of acetic acid was highest with *Ulva* sp., *G. vermiculophylla*, and *S. latissima* (63.4, 63.4, and 64.3%, respectively), and the lowest proportion was in the control (61.3%), which was not different from the remaining seaweeds (*L. ochroleuca* and *Gigartina* sp.). Conversely, the butyric acid proportion was highest in the control and *L. ochroleuca* and lowest with *Ulva* sp., *S. latissima, Gigartina* sp., and *G. vermiculophylla*. A tendency for a decrease in the propionic acid proportion with seaweeds was observed (*P* = 0.086), whereas the proportion of *iso*-valeric was highest and was significantly different from the control with *Gigartina* sp. When incubated with meadow hay, *L. ochroleuca* tended to decrease the *iso*-valeric proportion, while *Gigartina* sp. tended to increase the proportion of this VFA when incubated with corn silage (*P* = 0.068 for substrate and seaweed interaction; Fig. 1d).

**Figure 2:**
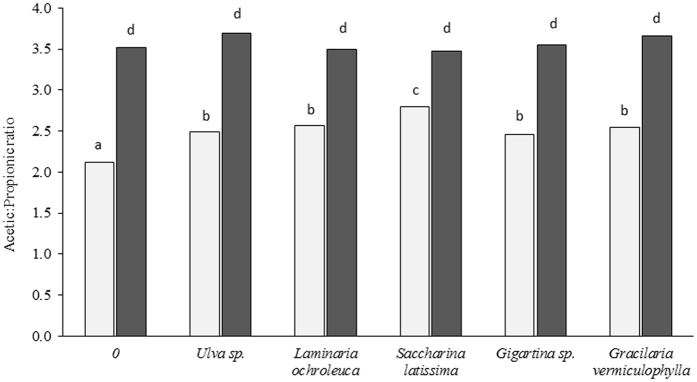
Effects of seaweed*inoculum interaction on acetic:propionic ratio after 24 h of *in vitro* incubation. 5% oil inoculum (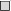), 0% oil inoculum (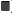). Mean values with different superscript letters were significantly different (*P* < 0.05).

The basal substrate did not alter the acetic:propionic ratio in the control, which was similar to that observed with the inclusion of seaweeds with corn silage, whereas meadow hay increased the ratio (Fig. 1e). The acetic:propionic ratio increased by 9% with seaweed inclusion compared to the control (*P* < 0.001; Table 4); no differences were observed among seaweed species. Additionally, the acetic:propionic ratio was affected by the interaction between substrate and seaweed (Fig. 1e) and between seaweed and inoculum (Fig. 2). Unadapted inoculum led to a similar and higher acetic:propionic ratio in the control and with seaweed inclusion, regardless of the species, compared to oil-adapted inoculum, the lowest ratio being detected in the control incubated with adapted inoculum (Fig. 2).

### Hydrogen balance

Hydrogen generated and consumed, percentage recovery and fermentation efficiency were significantly affected (*P* < 0.001; Table 2) by substrate and inoculum, but not by seaweed inclusion (*P* > 0.05; Table 3). Corn silage generated and consumed more hydrogen than meadow hay, the percentage of recovery being 36.6% and the fermentation efficiency 75.4%. Similarly, oil-adapted inoculum generated and consumed more hydrogen, but the recovery and the fermentation efficiencies were lower than those observed with unadapted inoculum.

## Discussion

The effects of five seaweed species (green, brown and red) either highly available on the Atlantic and Mediterranean coasts or produced in an IMTA system were evaluated in short-term *in vitro* rumen fermentation batches incubated at a high inclusion level (25% DM basis) with two different substrates (75% DM basis). In the IMTA system, the by-products (wastes) from one species (fish) are recycled to become inputs (fertilizers, food) for another (*e.g*., algae), resulting in the additional production of a marketable product with little or no additional input costs, a decrease in waste outputs from overall farming activities, and more environmentally sustainable farming. In this context, we evaluated the effects of seaweed inclusion and its interactions with basal diet on gas and methane production, VFA, hydrogen balance and NH_3_-N.

Feeding strategies to decrease gas emissions, particularly methane emissions, from livestock have focused on the manipulation of ruminal microbial populations and metabolism through the nutritional and biochemical properties of feeds. However, the decrease in methane production must be achieved with no or minimal adverse effects on overall rumen fermentation. In the present study, despite corn silage having decreased the fermentation pH below 6.0, which inhibits methanogen growth^17^, it increased gas and methane production, reflecting a greater extent of fermentation of this substrate relative to meadow hay. Rumen inoculum from cows adapted to a diet supplemented with 5% sunflower oil led to a decrease in gas and methane production. Dietary lipid supplementation constitutes a nutritional strategy to mitigate methane emissions, with differences in methanogenesis depending on the type of fat and its availability in the rumen^18^. Fatty acids may inhibit methane production by direct toxic effects on ruminal microorganisms and protozoa^19^ and indirectly on protozoa-associated methanogens^20^. Therefore, the decrease in methanogenesis might be attributed to a reduced abundance of archaea due to protozoan inhibition. Seaweeds have been evaluated for their effects on ruminal methane production. Although only a limited variety of seaweeds were assessed, some have shown a great potential to decrease methanogenesis (*e.g*., *Asparagopsis taxiformis*)^13^, while others have high nutritional value but lower antimethanogenic potential (*Chondrus cripus*, *Laminaria longicruris*, and *Fucus vesiculosus*^15^; *Spirogyra* and *Derbesia*^12^; and *Caulerpa taxifolia* and *Tarong polyculture*^11^). In our study, all seaweeds reduced total gas production (mL g^−1^ DM) after 24 h incubation, with *Gigartina* sp. promoting the greatest decrease, although chemical parameters were unable to explain this effect. Methane production (mL g^−1^ DM) was affected differently by different seaweeds. Green and red algae reduced methanogenesis, with *G. vermiculophylla* and *Gigartina* sp. having the most noticeable effects (38.2 and 35.8% reduction, respectively); brown seaweeds had no effect compared to the control. Red and brown algae exert more marked effects on methane production than green algae^11^,^12^,^13^. Indeed, the red *Asparagopsis* has potent antimethanogenic properties (more than 99% decrease in methane production) *in vitro* at 1% or 2% inclusion levels (organic matter, OM, basis)^13^,^14^. This effect on methanogenesis reduction has been suggested to be associated with seaweed secondary metabolites^12^,^13^,^14^,^15^. Indeed, seaweeds have developed a complexity and diversity of secondary compounds as a defence mechanism for survival in a highly competitive environment^21^. Red seaweeds are particularly rich with more than 1500 secondary metabolites of all classes, particularly halogenated compounds with bromine or chlorine^22^ that inhibit the methyl transfer reactions essential for methanogenesis^23^. Brown algae also possess a wealth of secondary metabolites (more than 1100 reported), in particular phlorotannins (polyphenols exclusive to brown algae), which exert an anti-microbial action, particularly on the widespread rumen cellulolytic bacterium *Fibrobacter succinogenes*^24^. Conversely, green seaweeds have the least variety of secondary metabolites, with fewer than 300 compounds found^25^. In addition to seaweed individual effects, an interaction between seaweeds and basal substrate was observed for methane production, with *Ulva* sp. and *Gigartina* sp. only decreasing methane production when incubated with corn silage and *G. vermiculophylla* decreasing methane production independently of the substrate used. Indeed, some studies suggest that regardless of the compound used to decrease methane emissions, the basal diet fed to the animal plays an important role in the effectiveness of the compound. For instance, the supplementation of *Oedogonium* (0.2 g OM basis) to different basal substrates (1 g OM basis) has been found to decrease methane at different rates, by nearly 40%^11^, 30%^12^ or 15%^13^, when Rhodes grass (107 g kg^−1^ CP, 672 g kg^−1^ NDF, DM basis), Finders grass (27.5 g kg^−1^ CP, 746 g kg^−1^ non-structural carbohydrates, DM basis) or Rhodes grass hay (66.9 g kg^−1^ CP, 766 g kg^−1^ carbohydrates, DM basis), respectively, was used as basal substrate. Machado *et al*.^13^ suggested that differences in the substrate used across different studies may have contributed to the variable *in vitro* antimethanogenic effect of *Oedogonium* sp., as high-protein substrates lead to lower gas and methane productions than low-quality fibrous substrates. Additionally, Machmuller *et al*.^26^ found that the decrease in methane production observed with myristic acid doubled when sheep consumed a concentrate (mean CP 167 g kg^−1^ DM) compared to when it consumed a forage-based diet (mean CP 139 g kg^−1^ DM). Conversely, O’Brien *et al*.^16^ found that the effectiveness of lauric, oleic, linoleic and linolenic acids and bromoethanesulfonate in reducing methane production was more pronounced when incubated with grass silage and barley grain (116 g CP kg^−1^ DM) than with perennial ryegrass (161 g CP kg^−1^ DM). In the present study, corn silage presented a CP content 39% higher than meadow hay, which could have partly contributed to the results observed. However, comparing our results to those in the literature is difficult because, to our knowledge, this is the first time that these five seaweeds have been studied and screened with contrasting substrates.

Volatile fatty acids are produced through the fermentation of dietary OM by the complex microbial ecosystem in the rumen. Volatile fatty acids are energy sources for maintenance and growth, propionic acid being a primary glycogenic precursor, butyric acid a lipogenic precursor of longer-chain fatty acids, and acetic acid a primary precursor of short- and medium-chain fatty acids^27^. The amount, type and rate of fermentation of dietary carbohydrates affect both the total amounts and proportions of individual VFAs formed and, ultimately, the amount of methane produced. In our study, corn silage increased total VFA production and decreased pH, reflecting again a higher extent of fermentation of this substrate relative to meadow hay. Additionally, corn silage (with 300 g kg^−1^ DM of starch) promoted higher propionic acid and lower acetic acid proportions than meadow hay. Diets rich in starch can decrease rumen pH, thus reducing fibre digestibility^28^ and promoting propionic acid production, whilst roughage-based diets promote acetic acid production^29^. Rumen inoculum from cows adapted to a diet supplemented with 5% sunflower oil led to a decrease in the acetic acid proportion and an increase in total VFA production and the propionic acid proportion. Acetic acid and butyric acid promote methane production, whilst propionic acid production can be considered a competitive pathway for hydrogen use in the rumen^30^. Methane production also decreased with oil-adapted rumen inoculum, suggesting that to compensate for the disruption of electron flow to methanogenesis, the rumen microbial population disposed of excess reducing equivalents by increasing the production of more reduced VFA, thus decreasing acetic acid production^3^,^31^. Fatty acids have a strong inhibitory effect on protozoa and cellulolytic bacteria^19^, while propionic acid-producing Gram-negative bacteria are not significantly inhibited^32^. A reduction in methane production thus shifts fermentation towards propionic acid production^33^. A slight decrease in the valeric acid proportion accompanying the decrease in methane production suggests a small shift in the fermentation pattern already seen in previous research with the addition of short-chain fatty acids^34^. Overall, seaweeds decreased methanogenesis, but for practical application, this reduction should have no or minimal negative effects on fermentation parameters, including the production of VFA.

In this study, the reduced fermentation suggested by the decreased total gas and methane production was not supported by total VFA production, which was unaffected by seaweed inclusion. With the non-significant differences between seaweed inclusion and the control on total VFA production, the reason for reduced gas and methane production remains unclear. The presence of bioactive compounds and the ability of the different classes of rumen microbes to efficiently use polysaccharides from the cell walls of the different seaweeds might contribute to explaining these results. Unlike terrestrial plants, seaweeds have complex polysaccharides in the cell wall structure, which greatly differ among seaweed classes and species^35^. Green algae are rich in soluble ulvans of the family of sulphated polysaccharides^36^. Conversely, brown algae are rich in laminarin, mannitol, alginic acid, fucoidans and, in only minor amounts, cellulose^37^, while carrageenans^38^, agars, and porphyran^39^ are the major matrix polysaccharides of red algae. Although ulvans are potentially hydrolysable to bioactive oligosaccharides^40^, ulvan lyases have only been isolated in marine environments^41^ and in *Proteobacteria* species found in soil^42^. Similarly, the hydrolysis of red algal galactans requires a range of enzymes predominantly encoded in the genomes of marine microbes but that are less frequent or even absent in the bacteria that hydrolyse polysaccharides from land plants^43^. Brown seaweed carbohydrates are hydrolysed by the rumen microbial population, producing methane and acetic acid^44^ (though to different extents). Indeed, Orpin *et al*.^45^ found that 13% of the culturable bacteria from seaweed-fed sheep grew on alginate, 71% on laminarin, 13% on fucoidan, and 99% on mannitol, whilst the percentages obtained from pasture-fed animals were significantly lower (2%, 32%, 0% and 0%, respectively). Differences in the ability to hydrolyse mannitol between seaweed-fed or grass-fed animals were also observed in other studies^46^.

The effects of seaweeds on total VFA production depended on the substrate used. The combination of *L. ochroleuca*, *Gigartina* sp., and *G. vermiculophylla* with corn silage had a negative effect on total VFA production, indicating that the type and quality of substrates influence the extent of the adverse effects on *in vitro* fermentation. Horn and Østgaard^47^ studied the anaerobic digestion of alginate from *Laminaria hyperborea* and reported that the production of extracellular polymer-degrading enzymes such as alginate lyase could be suppressed in the presence of easily usable alternative substrates. This could partly explain the decrease in total VFA production when *L. ochroleuca* was incubated with corn silage, as extra glucose can lead to a diauxic development with glucose as the preferred substrate and delayed initiation of alginate lyase activity^47^.

Seaweeds differently affected individual VFA proportions; only seaweeds from an IMTA system significantly increased the acetic acid proportion when compared to the control. No differences among seaweeds were observed regarding the propionic acid proportion, suggesting that reducing equivalents were not redirected to the production of this reduced fermentation product, not even with *Gigartina* sp., which promoted the lowest methane production. A significant interaction was observed for the acetic acid:propionic acid ratio, with seaweeds having a cumulative effect when incubated with meadow hay or with the oil-adapted inoculum. With the exception of *L. ochroleuca*, all other seaweeds decreased the butyric acid proportion, suggesting that the reducing equivalents spared from methanogenesis were not consumed during the formation of this fermentation product. With red seaweeds, some reducing equivalents may have been consumed via increased proportions of *iso*-valeric acid. The consumption of reducing equivalents might also have occurred during anabolic processes (*e.g*., cell growth, extracellular polysaccharide production)^48^ or through the reduction of carbon dioxide to acetic acid via acetogenesis^49^.

Methanogens are the main users of hydrogen within the rumen. The inhibition of methanogenesis can lead to the accumulation of excess reducing equivalents that can increase intracellular NADH/NAD, thus reducing overall fermentation efficiency by limiting the availability of oxidized cofactors required for glycolysis^31^ or leading to an increase in propionic acid or NH_3_-N production^2^. In this study, corn silage and oil-unadapted inoculum led to a high percentage of hydrogen recovery and fermentation efficiency. Although seaweed inclusion did not significantly affect hydrogen balance or fermentation efficiency, it increased NH_3_-N production, suggesting that reducing equivalents spared from methane production did not accumulate as reduced NADH. However, the ammonia accumulation must be interpreted with caution, as free ammonia may be both produced and assimilated by the rumen microbial population and can also reflect the higher CP content in seaweeds than in substrates.

In conclusion, this study demonstrated that *Ulva* sp., *L. ochroleuca*, *S. latissima*, *Gigartina* sp., and *G. vermiculophylla* strongly reduced methanogenesis when incubated at 25%, with *Gigartina* sp. promoting the lowest methane production. The studied seaweeds did not have any detrimental effects on *in vitro* rumen fermentation when compared to the control. However, the effects on methane and total VFA production depended on the substrate used. All seaweeds decreased methane production when incubated with meadow hay, but only *G. vermiculophylla* decreased it when incubated with corn silage. The combination of *L. ochroleuca*, *Gigartina* sp., and *G. vermiculophylla* with corn silage had a negative effect on total VFA production. The results suggest that seaweeds have the potential to be used as a feed ingredient in animal diets at relatively high levels as sources of macro-nutrients and bioactive compounds, with a beneficial effect on reduced methane emissions; however, the basal diet given to the animal must be considered. Moreover, longer-duration studies are required, particularly *in vivo*, to confirm the effectiveness of seaweeds as natural manipulators of ruminal methanogenesis and to screen them with different basal diets.

## Methods

### Seaweeds and basal substrates

Green macroalgae (*Ulva* sp.), brown macroalgae (*L. ochroleuca*, *S. latissima*) and red macroalgae (*Gigartina* sp., *G. vermiculophylla*) were studied. Seaweeds were harvested off the north coast of Portugal or produced in an IMTA system^50^ as described in Table 4. After collection, seaweed biomass was rinsed in freshwater to remove epiphytes, detritus, and sand and was transported to the laboratory and dried in a forced-air oven at 65 °C until a constant weight was achieved.

Meadow hay and corn silage were used as basal substrates in the *in vitro* incubations, after being dried for 48 h in a forced-air oven at 65 °C. Dried seaweeds, meadow hay and corn silage samples were ground to pass through a 1-mm screen and were stored at room temperature until incubation.

Ground (1 mm) samples of seaweeds and substrates used in the *in vitro* incubations were analysed for DM^51^, ash (ID 942.05)^51^, ether extract (EE; ID 920.39)^51^, Kjeldahl N (ID 954.01)^51^, NDF (with α-amylase and without sodium sulphite), acid detergent fibre (ADF) and ADL^52^,^53^. Crude protein was determined as Kjeldahl N × 6.25 for substrates and Kjeldahl N × 5.0 for seaweeds^54^. Neutral detergent fibre and ADL were expressed exclusive of residual ash. The starch content of corn silage was determined on finely ground samples with a 0.5-mm screen^55^. The GE of seaweeds and substrates were determined in an adiabatic bomb calorimeter (Werke C2000, IKA, Staufen, Germany). All chemical analyses were run in duplicate. Non-starch polysaccharides (NSP) were calculated by the difference between DM and the sum of ash, EE, CP, NDF and starch.

### Rumen inoculum and diet

Rumen contents were obtained from two adult Holstein cows, dry and not pregnant, fitted with a rumen cannula (10 cm diameter; Bar Diamond Inc., Parma, ID). Cows were housed at the Vairão Agricultural Campus of Abel Salazar Biomedical Sciences Institute, University of Porto (Vila do Conde, Portugal) and were handled in strict accordance with good animal practice as defined by national authorities and the European Union Directive 2010/63/EU. The experimental animal procedures were approved by the Local Animal Ethics Committee of ICBAS-UP, licensed by the Portuguese Directorate-General of Food and Veterinary Medicine (Direção Geral de Alimentação e Veterinária) of the Ministry for Agriculture and Sea (Ministério da Agricultura e do Mar, permit #FT2014DGV 046412 ICB), and conducted by trained scientists following FELASA category C recommendations. All methods and procedures were performed following the established guidelines from these institutions.

A single total mixed ration (TMR) was used to feed the cows supplemented with 0% or with 5% sunflower oil (Fula Puro Girassol, Sovena, Algés, Portugal). The TMR comprised 14 kg corn silage (the same used as substrate in the *in vitro* incubations; Table 1), 3 kg wheat straw (37 g kg^−1^ CP, 811 g kg^−1^, NDF, DM basis), and 2 kg commercial concentrate for dry cows (230 g kg^−1^ CP, 294 g kg^−1^ NDF, 187 g kg^−1^ starch, DM basis). Cows were fed twice a day, at 0930 and 1730 h, the daily amount of feed being offered equally in both meals. Animals had continuous access to fresh drinking water. After a two-week adaptation period to the diet, rumen contents were collected from the four quadrants of the rumen of each cow and placed in a 4 L pre-warmed (39 °C) thermal jug. At the laboratory, each ruminal digesta was homogenized, strained through 4 layers of linen cloth, and maintained at 39 °C under O_2_-free CO_2_. The length of time between collection of rumen contents and incubation never exceeded 60 min. After rumen inocula collection, the diet was exchanged between cows and another two-week adaptation period began for a new collection of ruminal contents.

### Rumen *in vitro* incubations

The effects of seaweed supplementation to meadow hay and corn silage on the ruminal fermentation parameters, total gas production and methane production were evaluated in short-term (24 h) batch incubations. To each basal diet, one of the five seaweeds was supplemented at 0% (control) and 25% of the total incubated DM. One part of strained ruminal fluid was diluted anaerobically into four parts of the medium described by Marten and Barnes^56^ and mixed at 39 °C under O_2_-free CO_2_. Twenty five millilitres of the buffered ruminal fluid was dispensed anaerobically into 125 mL serum bottles (Sigma-Aldrich Inc., St. Louis, MO) containing 250 mg DM of each experimental treatment, sealed with butyl rubber stoppers (Sigma-Aldrich Inc., St. Louis, MO), and incubated in a water bath at 39 °C. Fermentations were stopped after 24 h by cooling the bottles in an ice-slurry bath at 4 °C. Experimental treatments were incubated in duplicate per inoculum and per incubation, and batch incubations were replicated in two separate runs.

### Incubation media sampling and analysis

Bottles were warmed to 25 °C, and head-space gas volume was measured with a pressure transducer (Bailey & Mackey Ltd., Birmingham, UK) as described by Theodorou *et al*.^57^. The composition of the head-space gas was determined in 0.5 mL samples collected with a gas-tight syringe (SGE international PTY Ltd, Australia) by gas chromatography, using a GC-4000A (East & West Analytical Instruments, Inc, Beijing, China) equipped with a Shincarbon ST 100/120 micropacked column (Restek Corporation, Bellefonte, PA) and a thermal conductivity detector. The temperature was held at 100 °C in the injector, 180 °C in the detector and in the bridge, and 60 °C in the oven. Helium was used as the carrier gas at a flow rate of 23 mL min^−1^. The analyses were performed in duplicate. An external standard with known composition (60% CO_2_, 25% N_2_, 10% CH_4_ and 5% H_2_; Air Liquide, Lda., Algés, Portugal) was used to identify and quantify gas peaks. Methane production was calculated according to Lopez and Newbold^58^, using CO_2_ as reference element of the gas mixture.

The pH of each bottle was measured immediately after gas sampling. Fermentation media contents were sub-sampled for the analysis of VFA and NH_3_-N. For VFA analyses, 0.25 mL of 25% ortho-phosphoric acid solution with internal standard (16 mM 3-methyl valeric acid; Sigma-Aldrich Inc., St. Louis, MO) was added to 1 mL of fermentation medium in a microcentrifuge tube, mixed and centrifuged at 19,800 × *g* at 4 °C for 15 min. The supernatant was filtered through a 25 mm polyethersulfone syringe filter (0.45 μm pore size; VWR International - Material de Laboratório, Lda., Carnaxide, Portugal) and stored at 4 °C until chromatographic analysis. Volatile fatty acids were analysed by gas chromatography using a Shimadzu GC-2010 Plus (Shimadzu Corporation, Kyoto, Japan) equipped with a capillary column (HP-FFAP, 30 m × 0.25 mm × 0.25 μm; Agilent Technologies, Santa Clara, CA), and a flame ionization detector. Injector and detector temperatures were held at 260 °C. The oven temperature started at 80 °C for 1 min, increased at 20 °C min^−1^ to 120 °C, then increased at 6 °C min^−1^ to 205 °C and finally increased at 20 °C min^−1^ to 240 °C. Helium was used as a carrier gas at a flow rate of 0.86 mL min^−1^. The injection volume was 1 μL and the split of 50:1. Volatile fatty acids were quantified with the internal standard (3-methyl valeric acid) and identified by comparison of retention times with a standard (Volatile Free Acid Mix, Sigma-Aldrich Inc., St. Louis, MO).

For NH_3_-N analysis, 5 mL of fermentation medium was added to 5 mL of 0.2 N HCl solution and steam-distilled (Vapodest 40, distiller unit, C. Gerhardt GmbH & Co. KG, Germany). N content was determined through titration (ID 954.01)^51^.

The reducing equivalents generated (expressed as μmol H_2_ mL^−1^ fermentation media) were estimated as 2 equiv. acetic acid + 1 equiv. propionic acid + 4 equiv. butyric acid + 2 equiv. valeric acid + 2 equiv. *iso*-valeric acid. The reducing equivalents (μmol H_2_ mL^−1^ fermentation media) consumed were estimated as 2 equiv. propionic acid + 2 equiv. butyric acid + 1 equiv. valeric acid + 4 equiv. methane^59^. Fermentation efficiency was calculated as (0.62 acetic acid + 1.09 propionic acid + 0.78 butyric acid)/(acetic acid + propionic acid + butyric acid) × 100 and is based on the heats of combustion of glucose in the respective VFA^60^.

### Statistical analysis

All data were analysed using the MIXED procedure of the SAS software program (2002; version 9.1, SAS Institute Inc., Carry, NC). The statistical model included the fixed effect of seaweed, substrate, rumen inoculum, and all the interactions between main effects, the random effect of the trial, and the random residual error. Effects were considered significant when *P* ≤ 0.05 and a trend when 0.05 < *P* ≤ 0.10. When the interactions had a non-significant effect or tendency (*P* > 0.10), they were removed from the model. The linear relationships between the chemical composition of the seaweeds and gas and methane production were evaluated using the REG procedure of the SAS software program (2002; version 9.1, SAS Institute Inc., Carry, NC).

## Additional Information

**How to cite this article**: Maia, M. R. G. *et al*. The Potential Role of Seaweeds in the Natural Manipulation of Rumen Fermentation and Methane Production. *Sci. Rep*. **6**, 32321; doi: 10.1038/srep32321 (2016).

## Figures and Tables

**Table 1:** Proximate composition and energy contents of seaweeds and chemical compositions of meadow hay and corn silage used as substrates in the *in vitro* incubations.

	DM	Ash	EE	CP	NDF	ADF	ADL	Starch	NSP	GE
		(g kg^−1^ DM)								(MJ kg^−1^ DM)
*Ulva* sp.	ND	250	3.20	124	335	132	79.5	ND	257	10.4
*Laminaria ochroleuca*	197	266	4.88	97.6	198	183	97.5	ND	409	11.2
*Saccharina latissima*	ND	171	7.87	143	71.1	48.2	14.8	ND	571	12.8
*Gigartina* sp.	212	348	3.22	119	322	82.9	16.0	ND	178	9.51
*Gracilaria vermiculophylla*	ND	278	2.55	167	136	40.1	10.4	ND	375	11.6
Meadow hay	723	68.1	16.2	53.8	565	342	39	ND	297	18.0
Corn silage	493	29.8	28.8	74.6	377	222	32	300	190	18.8

DM, dry matter; EE, ether extract; CP, crude protein; NDF, neutral detergent fibre; ADF, acid detergent fibre; ADL, acid detergent lignin; NSP, non-starch polysaccharides; GE, gross energy; ND, not determined.

**Table 2:** Effects of substrate and ruminal inoculum on gas production and composition, pH, ammonia-N (NH_3_-N), and volatile fatty acids (VFA) from *in vitro* 24-h batch incubations.

	Substrate		SEM	*P*	Inoculum		SEM	*P*
	Hay	Corn silage			5% oil	0% oil		
Gas, mL	16.0	24.6	7.92	<0.001	15.1	25.5	7.92	<0.001
Gas, mL g^−1^ DM	68.8	103.3	33.56	<0.001	64.1	108.0	33.56	<0.001
Methane, mL	0.239	0.474	0.073	<0.001	0.249	0.464	0.073	<0.001
Methane, mL g^−1^ DM	1.027	1.987	0.3111	<0.001	1.051	1.963	0.3110	<0.001
pH	6.10	5.91	0.071	<0.001	5.98	6.03	0.071	0.056
NH_3_-N, mg g^−1^ DM	4.33	4.19	0.661	0.406	3.55	4.96	0.661	<0.001
Total VFA, mmol g^−1^ DM	2.94	3.59	0.196	<0.001	3.74	2.79	0.196	<0.001
Acetic acid, %	64.8	61.2	2.57	<0.001	62.8	63.2	2.57	<0.001
Propionic acid, %	22.2	23.7	4.66	<0.001	26.9	18.9	4.66	<0.001
*Iso*-butyric acid, %	0.73	1.18	0.278	<0.001	1.13	0.78	0.278	<0.001
Butyric acid, %	9.1	10.4	1.11	<0.001	6.3	13.2	1.11	<0.001
*Iso*-valeric acid, %	1.18	1.31	0.043	<0.001	1.28	1.21	0.043	0.042
Valeric acid, %	1.52	1.48	0.410	0.530	1.31	1.70	0.410	<0.001
Caproic acid, %	0.439	0.605	0.2334	<0.001	0.178	0.866	0.2334	<0.001
Acetic:propionic acid ratio	3.18	2.88	0.683	<0.001	2.50	3.57	0.683	<0.001
H_2_ generated, mmol L^−1^	53.5	67.0	5.50	<0.001	72.4	48.0	5.50	<0.001
H_2_ consumed, mmol L^−1^	17.3	23.5	0.69	<0.001	22.6	18.2	0.69	<0.001
Recovery, %	33.5	36.6	4.73	<0.001	31.7	38.3	4.73	<0.001
Fermentation efficiency, %	74.3	75.4	2.00	<0.001	73.2	76.5	2.00	<0.001

Seaweed*substrate significant for methane (mL g^−1^ DM; *P* = 0.002), total VFA (mmol L^−1^; *P* = 0.092), *iso*-valeric acid (%, *P* = 0.068), acetic:propionic ratio (*P* = 0.031), and N-NH_3_ (mg g^−1^ DM; P <0.001). Seaweed*inoculum significant for acetic:propionic ratio (*P* < 0.001). Substrate*inoculum significant for methane (mL g^−1^ DM; *P* = 0.046), N-NH_3_ (mg g^−1^ DM; *P* = 0.006), acetic acid (%, *P* = 0.032), propionic acid (%, *P* < 0.001), *iso*-butyric acid (%, *P* = 0.038), butyric acid (%, *P* < 0.001), caproic acid (%, *P* < 0.001), and acetic:propionic ratio (*P* < 0.001). Fermentation efficiency (%, P = 0.004). Recovery (%, P = 0.026).

**Table 3:** Effects of seaweed on gas production and composition, pH, ammonia-N (NH_3_-N), and volatile fatty acids (VFA) from *in vitro* 24-h batch incubations.

	Seaweed						SEM	*P*
	Control	*Ulva* sp.	*Laminaria ochroleuca*	*Saccharina latissima*	*Gigartina* sp.	*Gracilaria vermiculophylla*		
Gas, mL	23.7^a^	20.3^c^	21.2^c^	21.1^c^	16.0^b^	19.6^c^	7.94	<0.001
Gas, mL g^−1^ DM	100.5^a^	86.2^c^	89.5^c^	89.9^c^	67.5^b^	82.5^c^	33.65	<0.001
Methane, mL	0.413^a^	0.308^b^	0.472^a^	0.425^a^	0.266^b^	0.255^b^	0.0780	<0.001
Methane, mL g^−1^ DM	1.754^a^	1.301^b^	1.984^a^	1.813^a^	1.117^b^	1.072^b^	0.3322	<0.001
pH	5.94	6.03	6.02	5.99	6.02	6.02	0.076	0.306
NH_3_-N, mg g^−1^ DM	3.43^a^	4.47^b^	3.52^a,d^	3.86^a,d^	6.07^c^	4.18^b,d^	0.681	<0.001
Total VFA, mmol g^−1^ DM	3.38^ab^	3.20^a^	3.03^a^	3.60^b^	3.21^a^	3.20^a^	0.220	0.033
Acetic acid, %	61.3^a^	63.4^b^	62.8^ab^	64.3^b^	62.8^ab^	63.4^b^	2.61	0.023
Propionic acid, %	24.0	22.8	22.6	22.4	23.2	22.7	4.67	0.086
*Iso*-butyric acid, %	0.832	0.997	1.033	0.882	0.994	0.983	0.2834	0.231
Butyric acid, %	10.4^b^	9.6^ac^	10.0^bc^	9.3^a^	9.6^a^	9.5^a^	1.11	<0.001
*Iso*-valeric acid, %	1.25^ab^	1.24^ab^	1.18^a^	1.12^a^	1.39^c^	1.31^bc^	0.056	<0.001
Valeric acid, %	1.60	1.45	1.57	1.44	1.48	1.49	0.415	0.599
Caproic acid, %	0.551	0.484	0.650	0.469	0.492	0.486	0.2377	0.176
Acetic:propionic acid ratio	2.82^a^	3.09^b^	3.03^b^	3.14^b^	3.00^b^	3.10^b^	0.684	0.002
H_2_ generated, mmol L^−1^	62.4	58.9	56.0	65.4	59.3	59.4	5.84	0.118
H_2_ consumed, mmol L^−1^	21.4	22.2	19.6	19.2	23.5	20.2	1.01	0.161
Recovery, %	36.9	34.8	34.7	33.6	35.0	35.1	4.79	0.233
Fermentation efficiency, %	75.5	74.8	74.7	74.5	74.9	74.8	2.01	0.280

**Table 4:** Species and harvesting area and year of studied seaweeds.

Species	Class	Harvesting area	Harvesting year
*Ulva* sp.	Green	Cultivated	2012
*Laminaria ochroleuca*	Brown	Praia da Amorosa, Viana do Castelo (41° N, 8° W)	2013
*Saccharina latissima*	Brown	Cultivated	2013
*Gigartina* sp.	Red	Praia da Amorosa, Viana do Castelo (41° N, 8° W)	2013
*Gracilaria vermiculophylla*	Red	Cultivated	2012

## References

[b1] Williams A. G. & Coleman G. S. In The Rumen Microbial Ecosystem (eds Hobson P. N. & Stewart C. S.), 73–139 (Springer Netherlands, 1997).

[b2] Morgavi D. P., Forano E., Martin C. & Newbold C. J. Microbial ecosystem and methanogenesis in ruminants. animal 4, 1024–1036 (2010). 22444607 10.1017/S1751731110000546

[b3] Van Nevel C. J. & Demeyer D. I. Control of rumen methanogenesis. Environ. Monit. Assess. 42, 73–97 (1996). 24193494 10.1007/BF00394043

[b4] Patra A. K. Enteric methane mitigation technologies for ruminant livestock: a synthesis of current research and future directions. Environ. Monit. Assess. 184, 1929–1952 (2011). 21547374 10.1007/s10661-011-2090-y

[b5] Balasse M., Tresset A., Dobney K. & Ambrose S. H. The use of isotope ratios to test for seaweed eating in sheep. J. Zool. 266, 283–291 (2005).

[b6] Makkar H. P. S. . Seaweeds for livestock diets: A review. Anim. Feed Sci. Technol. 212, 1–17 (2016).

[b7] Kumar C. S., Ganesan P., Suresh P. V. & Bhaskar N. Seaweeds as a source of nutritionally beneficial compounds - A review. J. Food Sci. Tech. Mys. 45, 1–13 (2008).

[b8] Ramnani P. . *In vitro* fermentation and prebiotic potential of novel low molecular weight polysaccharides derived from agar and alginate seaweeds. Anaerobe 18, 1–6 (2012). 21924371 10.1016/j.anaerobe.2011.08.003

[b9] Machado L., Kinley R. D., Magnusson M., Nys R. & Tomkins N. W. The potential of macroalgae for beef production systems in Northern Australia. J. Appl. Phycol. 27, 2001–2005 (2015).

[b10] Jiménez-Escrig A. & Sánchez-Muniz F. J. Dietary fibre from edible seaweeds: Chemical structure, physicochemical properties and effects on cholesterol metabolism. Nutr. Res. 20, 585–598 (2000).

[b11] Dubois B. . Effect of Tropical Algae as Additives on Rumen *in vitro* Gas Production and Fermentation Characteristics Am. J. Plant Sci. 4, 34–43 (2013).

[b12] Machado L., Magnusson M., Paul N. A., Nys R. & Tomkins N. Effects of marine and freshwater macroalgae on *in vitro* total gas and methane production. PLoS One 9, e85289 (2014). 24465524 10.1371/journal.pone.0085289 PMC3898960

[b13] Machado L. . Dose-response effects of *Asparagopsis taxiformis* and *Oedogonium* sp. on *in vitro* fermentation and methane production. J. Appl. Phycol. 28, 1443–1452 (2015).

[b14] Kinley R. D., de Nys R., Vucko M. J., Machado L. & Tomkins N. W. The red macroalgae *Asparagopsis taxiformis* is a potent natural antimethanogenic that reduces methane production during *in vitro* fermentation with rumen fluid. Anim. Prod. Sci. 56, 282–289 (2016).

[b15] Kinley R. D. & Fredeen A. H. *In vitro* evaluation of feeding North Atlantic stormtoss seaweeds on ruminal digestion. J. Appl. Phycol. 27, 2387–2393 (2015).

[b16] O’Brien M., Navarro-Villa A., Purcell P. J., Boland T. M. & O’Kiely P. Reducing *in vitro* rumen methanogenesis for two contrasting diets using a series of inclusion rates of different additives. Anim. Prod. Sci. 54, 141–157 (2014).

[b17] Van Kessel J. A. S. & Russell J. B. The effect of pH on ruminal methanogenesis. FEMS Microbiol. Ecol. 20, 205–210 (1996).

[b18] Boadi D., Benchaar C., Chiquette J. & Massé D. Mitigation strategies to reduce enteric methane emissions from dairy cows: Update review. Can. J. Anim. Sci. 84, 319–335 (2004).

[b19] Zhang C. M. . Effect of octadeca carbon fatty acids on microbial fermentation, methanogenesis and microbial flora *in vitro*. Anim. Feed Sci. Technol. 146, 259–269 (2008).

[b20] Newbold C. J., Lassalas B. & Jouany J. P. The importance of methanogens associated with ciliate protozoa in ruminal methane production *in vitro*. Lett. Appl. Microbiol. 21, 230–234 (1995). 7576513 10.1111/j.1472-765x.1995.tb01048.x

[b21] Paul N. A., Nys R. & Steinberg P. D. Seaweed–herbivore interactions at a small scale: direct tests of feeding deterrence by filamentous algae. Mar. Ecol. Prog. Ser. 323, 1–9 (2006).

[b22] Blunt J. W. . Marine natural products. Nat. Prod. Rep. 24, 31–86 (2007). 17268607 10.1039/b603047p

[b23] Liu H., Wang J., Wang A. & Chen J. Chemical inhibitors of methanogenesis and putative applications. Appl. Microbiol. Biotechnol. 89, 1333–1340 (2011). 21193988 10.1007/s00253-010-3066-5

[b24] Wang Y., Alexander T. W. & McAllister T. A. *In vitro* effects of phlorotannins from *Ascophyllum nodosum* (brown seaweed) on rumen bacterial populations and fermentation. J. Sci. Food Agric. 89, 2252–2260 (2009).

[b25] Maschek J. A. & Baker B. J. In Algal Chemical Ecology (ed Amsler C. D.), 1–24 (Springer-Verlag Berlin Heidelberg, 2008).

[b26] Machmuller A., Machmuller A., Soliva C. R. & Kreuzer M. Methane-suppressing effect of myristic acid in sheep as affected by dietary calcium and forage proportion. Br. J. Nutr. 90, 529–540 (2003). 13129458 10.1079/bjn2003932

[b27] Bergman E. N. Energy contributions of volatile fatty acids from the gastrointestinal tract in various species. Physiol. Rev. 70, 567–590 (1990). 2181501 10.1152/physrev.1990.70.2.567

[b28] Hoover W. H. Chemical factors involved in ruminal fiber digestion. J. Dairy Sci. 69, 2255–2261 (1986). 10.3168/jds.S0022-0302(86)80724-X 3027148

[b29] Johnson K. A. & Johnson D. E. Methane emissions from cattle. J. Anim. Sci. 73, 2483–2492 (1995). 8567486 10.2527/1995.7382483x

[b30] Moss A. R., Jouany J.-P. & Newbold J. Methane production by ruminants: its contribution to global warming. Ann. Zootech. 49, 231–253 (2000).

[b31] Miller T. L. I. E., Ferdinand Enke Verlag, Berlin, pp. 317–331. in Ruminant physiology: digestion, metabolism, growth and reproduction (ed Engelhardt W. V., Leonhard-Marek S., Breves G., Giesecke D.), 317–331 (Ferdinand Enke Verlag, 1995).

[b32] Van Nevel C. J. & Demeyer D. I. In The Rumen Ecosystem (ed Hobson P. N.), 387–443 (Elsevier Science Publishers Ltd, 1988).

[b33] McAllister T. A., Cheng K. J., Okine E. K. & Mathison G. W. Dietary, environmental and microbiological aspects of methane production in ruminants. Can. J. Anim. Sci. 76, 231–243 (1996).

[b34] Zelenák I., Jalc D., Kmet V. & Siroka P. Influence of diet and yeast supplement on *in vitro* ruminal characteristics. Anim. Feed Sci. Technol. 49, 211–221 (1994).

[b35] Pereira L., Amado A. M., Critchley A. T., van de Velde F. & Ribeiro-Claro P. J. A. Identification of selected seaweed polysaccharides (phycocolloids) by vibrational spectroscopy (FTIR-ATR and FT-Raman). Food Hydrocoll. 23, 1903–1909 (2009).

[b36] Domozych D. S. . The Cell Walls of Green Algae: A Journey through Evolution and Diversity. Front. Plant Sci. 3, 82 (2012). 22639667 10.3389/fpls.2012.00082 PMC3355577

[b37] Sterner M. & Edlund U. Multicomponent fractionation of *Saccharina latissima* brown algae using chelating salt solutions. J. Appl. Phycol., 1–14 (2016). 10.1007/s10811-015-0785-0 PMC4947094 27471344

[b38] Michel G., Nyval-Collen P., Barbeyron T., Czjzek M. & Helbert W. Bioconversion of red seaweed galactans: a focus on bacterial agarases and carrageenases. Appl. Microbiol. Biotechnol. 71, 23–33 (2006). 16550377 10.1007/s00253-006-0377-7

[b39] Correc G., Hehemann J.-H., Czjzek M. & Helbert W. Structural analysis of the degradation products of porphyran digested by *Zobellia galactanivorans* [beta]-porphyranase A. Carbohyd. Polym. 83, 277–283 (2011).

[b40] Andrieux C. . *Ulva lactuca* is poorly fermented but alters bacterial metabolism in rats inoculated with human fecal flora from methane and non-methane producers. J. Sci. Food Agric. 77, 25–30 (1998).

[b41] Barbeyron T. . *Persicivirga ulvanivorans* sp. nov., a marine member of the family Flavobacteriaceae that degrades ulvan from green algae. Int. J. Syst. Evol. Microbiol. 61, 1899–1905 (2011). 20833882 10.1099/ijs.0.024489-0

[b42] Collén P. N., Sassi J.-F., Rogniaux H., Marfaing H. & Helbert W. Ulvan lyases isolated from the Flavobacteria *Persicivirga ulvanivorans* are the first members of a new polysaccharide lyase family. J. Biol. Chem. 286, 42063–42071 (2011). 22009751 10.1074/jbc.M111.271825 PMC3234910

[b43] Hehemann J. H. . Transfer of carbohydrate-active enzymes from marine bacteria to Japanese gut microbiota. Nature 464, 908–912 (2010). 20376150 10.1038/nature08937

[b44] Williams A. G., Withers S. & Sutherland A. D. The potential of bacteria isolated from ruminal contents of seaweed-eating North Ronaldsay sheep to hydrolyse seaweed components and produce methane by anaerobic digestion *in vitro*. Microb. Biotechnol. 6, 45–52 (2013). 23170956 10.1111/1751-7915.12000 PMC3815384

[b45] Orpin C. G., Greenwood Y., Hall F. J. & Paterson I. W. The rumen microbiology of seaweed digestion in Orkney sheep. J. Appl. Bacteriol. 58, 585–596 (1985). 4030526 10.1111/j.1365-2672.1985.tb01715.x

[b46] Ahmed S., Minuti A. & Bani P. *In vitro* rumen fermentation characteristics of some naturally occurring and synthetic sugars. Ital. J. Anim. Sci. 12, 359–365 (2013).

[b47] Horn S. J. & Østgaard K. Alginate lyase activity and acidogenesis during fermentation of *Laminaria hyperborea*. J. Appl. Phycol. 13, 143–152 (2001).

[b48] Russell J. B. Strategies that ruminal bacteria use to handle excess carbohydrate. J. Anim. Sci. 76, 1955–1963 (1998). 9690652 10.2527/1998.7671955x

[b49] Anderson R. C. . Effect of nitroethane, dimethyl-2-nitroglutarate and 2-nitro-methyl-propionic acid on ruminal methane production and hydrogen balance *in vitro*. Biores. Technol. 101, 5345–5349 (2010). 10.1016/j.biortech.2009.11.108 20194018

[b50] Domingues B., Abreu M. & Sousa-Pinto I. On the bioremediation efficiency of *Mastocarpus stellatus* (Stackhouse) Guiry, in an integrated multi-trophic aquaculture system. J. Appl. Phycol. 27, 1289–1295 (2015).

[b51] AOAC. Official Methods of Analysis. *15th ed. Association of Official Analytical Chemists*, Arlington, Vo. (1990).

[b52] Van Soest P. J., Robertson J. B. & Lewis B. A. Methods for dietary fiber, neutral detergent fiber, and nonstarch polysaccharides in relation to animal nutrition. J. Dairy Sci. 74, 3583–3597 (1991). 1660498 10.3168/jds.S0022-0302(91)78551-2

[b53] Robertson J. B. & Van Soest P. J. In The Analysis of Dietary Fiber in Food (eds James W. P. T. & Theander O.) 123–158 (Marcel Dekker Inc., 1981).

[b54] Angell A. R., Mata L., Nys R. & Paul N. A. The protein content of seaweeds: a universal nitrogen-to-protein conversion factor of five. J. Appl. Phycol. 28, 511–524 (2016).

[b55] Salomonsson A. C., Theander O. & Westerlund E. Chemical characterization of some Swedish cereal whole meal and bran fractions. Swed. J. Agr. Res. 14, 111–117 (1984).

[b56] Marten G. C. & Barnes R. F. Prediction of energy digestibility of forages with *in vitro* rumen fermentation and fungal enzyme systems. *Standardization of analytical methodology for feeds: Proceedings of a workshop held in Ottawa, Canada. 12–14 March 1979. Ottawa, Ont. IDRC*. (1980).

[b57] Theodorou M. K., Williams B. A., Dhanoa M. S., McAllan A. B. & France J. A simple gas production method using a pressure transducer to determine the fermentation kinetics of ruminant feeds. Anim. Feed Sci. Technol. 48, 185–197 (1994).

[b58] Lopez S. & Newbold C. J. In Measuring Methane Production From Ruminants (eds HarinderP S. Makkar & PhilipE Vercoe) Ch. 1, 1–13 (Springer, Netherlands, 2007).

[b59] Demeyer D. I. In Rumen Microbial Metabolism and Ruminant Digestion (ed Jouany J. P.) 217–237 (INRA Editions, 1991).

[b60] Chalupa W. Manipulating rumen fermentation. J. Anim. Sci. 46, 585–599 (1977).

